# Hospital and Institutional News

**Published:** 1918-03-30

**Authors:** 


### HOSPITAL AND INSTITUTIONAL NEWS.

### A FINANCIAL POLICY FOR LEEDS GENERAL INFIRMARY.

IN consequence of the deficit of £14,670 on the year's work of Leeds General Infirmary, Mr. Charles Lupton has made three suggestions, the adoption of which alone he believes can secure the financial future of the institution. He proposes that the well-to-do citizens of Leeds should be asked to contribute a capital sum. He also suggests that the workpeople should be invited to provide an additional weekly sum, in which case, if they wished, the extra money could be divided fairly among the General Infirmary and other institutions. The third proposal is twofold. It concerns the patients, who are to receive "stronger pressure" than before to pay for part, at least, of the cost of their keep; and, further, it will invite all patients who come from a distance greater than twenty miles to contribute a guinea a week towards their maintenance. We doubt whether the third proposal will raise sufficient money to justify the making of patients' payments a more or less obligatory and formal thing. Until a system of graduated payments is introduced the bringing of pressure to bear on patients is not likely to be very satisfactory. We would rather suggest that the proposed invitation to the workpeople to raise an additional weekly sum should be accompanied by an equally strong appeal to their employers to contribute not a casual cheque, but a definite sum per head of the number of men in their employment. The employers' associations have embraced this scheme in North Staffordshire. What reason is there to suppose that they will be less public-spirited in Leeds?

### APPLYING THE POLICY ALL ROUND.

THIS proposal to the employers should be quite separate from the general appeal to the well-to-do. But the employers should be invited to apply to themselves the methods which they have encouraged in their workpeople. As to the patients from outlying districts, we think that the districts rather than the patients should be subject to scrutiny and method. A series of district funds should be started in every area where the number and cost of patients sent to Leeds General Infirmary is out of all relation to the money contributed from the neighbourhood. A voluntary hospital is supported by the public, and if every section which the hospital serves is so organised as to provide its fair quota, such a big deficit as that at Leeds will soon be swept away. What the workpeople have been invited to do, their employers and the outlying districts should be invited to do also. It will be unfair to rely too much on the workpeople or the patients alone.

### MR. HODGE ON CHARLES DICKENS.

MR. JOHN HODGE, the Minister of Pensions, in a recent speech at Worcester, complained that he had authority without power and that the Treasury's sanction had to be sought on every occasion when the solution of any problem involved, as it generally does, some expense, even that of an extra 5s. a week to a typist. The Pensions Ministry, he added, was hampered by every Department. The extent of this embarrassment Mr. Hodge suggested very vividly by remarking that he used to think Dickens' description of the Circumlocution Office very much overdrawn, but that was before he himself became a Minister. Since he had become the head of a Government Department he had come to the conclusion that Dickens was right. There was too much red tape. There were too many delays; and when the Ministry was formed no one had realised what a huge concern it was going to become. In a few months the administration would be in full working order. He reminded his audience that a man's pension was fixed by his disability alone, and that doctors had no right to ask men what their work and wages were, men should refuse to answer such questions and should state that their authority for refusing was the Ministry of Pensions. He hoped shortly to have civilian wards for the discharge of men. The influence of Dickens has not been wasted.

### OFFICERS AND HOSPITAL STOPPAGES.

To improve the conditions of half-pay officers, and to amend the regulations in respect of hospital stoppages, a Royal Warrant has been issued as an Army Order. This lays down that from October 1917 and for the rest of the war the rate of half-pay payable to officers shall in no case be less than 5s. a day. From March 20, 1918, till the end of the war no stoppage shall be made from an officer's pay in respect of admission to hospital on account of illness clearly attributable to military service. The second provision secures to the officer of right that advantage which was previously in the discretion of his commanding officer.

### THE BRIGHTON PAVILION HOSPITAL'S LIBRARY.

FROM his appeal for works of a higher literary and educational value than the run of modern fiction with which the patients' library of the Brighton Pavilion Hospital is stocked, it seems that Colonel Maurice, the O.C., has leisure, after the recent disturbances at the hospital, to look after the higher needs of his patients. The capable administrator can be known from the military martinet by the readiness with which, once discipline has been secured, the former seeks to satisfy the legitimate and higher needs of the men under his command. We hope, therefore, that the request for presents of standard works, biography, works of travel, and the more ambitious quarterlies will meet with a ready response. The desire for these has been expressed, and that desire proves again the mixture of classes from which the new armies are drawn. It suggests, too, the possibility of a *Pavilion Hospital Gazette*. If the recent exuberance of energy can be directed into such a channel, an original and diverting magazine should result; and, since Colonel

Maurice has previous experience of the conduct of such a military paper, we hope that it may soon be forthcoming.

### EDUCATIONAL LECTURES FOR THE WOUNDED.

Some weeks ago several wounded men at the Great Northern Central Hospital made up their minds to organise lectures on educational subjects, chiefly technical. Private Mallet, who, before volunteering early in 1915, was a student in training as a teacher of handicraft at the L.C.C. Shoreditch Technical Institute, approached, on behalf of these men, the hospital authorities, and through Principal S. Hicks, some members of the staff of the Institute. At a meeting held at the hospital, the men stated very clearly what they felt they needed. Some few want books and guidance in taking up again and continuing their pre-war studies in such subjects as industrial chemistry, mathematics, accountancy, education, etc. Some asked for one or two lectures on gardening or agriculture; others are anxious to know something of the training that might be given them in boot-making, cabinet-making, wood-carving, picture-frame-making, bookbinding, etc. Many are interested in commercial work, others in architecture, while some asked for guidance in the study of English literature. Mr. Harris, chief librarian at the Islington Central Library, has kindly offered his services and expert advice in supplying standard technical books on loan to soldiers, and a course of lectures on many of the above subjects will be begun at once. Mr. P. A. Wells, head of the Cabinet-making Department of the L.C.C. Shoreditch Technical Institute, began the series by giving a lantern lecture on "Furniture" on Friday, March 8. The Principal of the Institute will give a lecture on the "Fisher Education Bill and the Need for Technical Teachers." We have only to remember that the Greek word for "leisure" is almost a synonym for the modern word "school" to understand the virtue of this departure. Time is the first essential, and the comic-song has too long been the beginning and the end of the "re-creation" of a wounded man in hospital. We are glad to note that the authorities of the Great Northern Central Hospital are inviting to these lectures soldier out-patients, discharged men, and convalescent soldiers from other hospitals.

### THE INIQUITIES OF THE VOTING SYSTEM.

The illustrated pamphlet entitled "An Afternoon Meeting, or How to Work a Case," which has been published as an appeal on behalf of the Royal Hospital for Incurables, Putney Heath, is a naked and unashamed exposure of the iniquities of the voting system. It will be a wonder if, after reading it, anyone consents to subscribe to a voting charity again. Under the guise of a drawing-room meeting over which a member of Parliament presides, which a bishop attends, and a local organiser addresses on behalf of a case which needs a thousand more votes to secure election to the charity, the whole dreary business of collecting votes is described till the demoralising farce is ended by the suggestion of an egregious officer of the charity that, after all, the shortest cut out of the mess is for those present, instead of soliciting further votes, to put up the necessary money themselves to purchase by subscription the number of votes required. The bland complacence of the secretary at converting the meeting to this view and at thereby having "netted" a considerable sum of money for his institution is quite appalling. It shows how men and women of original decency can be corrupted by habit into countenancing a procedure which involves the destruction of all public spirit in themselves, and reduces the voluntary system to a set of shifts which men in their right minds would be ashamed to foster. The voting system is merely the survival of petty patronage in its last stage of selling benefits for money. It involves nameless suffering on the waiting helpless cases, who often seek votes in vain for years. The law's delay is nothing compared with it, and modern hospital finance has proved that the sale of patronage is quite negligible as a foundation for income, besides degrading everyone who comes into contact with it. Some customs are so rotten that they survive in a kind of living death. Because no one defends them, they seem immune to attack or even ridicule. But if anything could destroy the voting system, which is a stain on any institution (however otherwise well managed) which adopts it, this account of a typical afternoon meeting should do so. Unfortunately the writer lacks a sense of humour, but of the kind of humour which we call unconscious, because it is exhibited in a guise of earnestness, there is sufficient. The result is an appalling sense of the fatuities committed daily in the name of charity, and of the worst evil which is still taken as a matter of course in some modern institutional affairs.

### THE OPPORTUNITY AT KINGSTON.

Mr. V. Knapp, the honorary treasurer of the Victoria Hospital, Kingston, in pointing out that there was a deficiency on the current account, apart from the sums received and ear-marked for investment, and the subscription list was falling, mentioned that the majority of the 50 odd extra patients who were treated last year, came from the munition works in the town. He thought that the employers might therefore be approached for liberal contributions. That is certainly reasonable, but we hope that Mr. Knapp and Mr. R. W. Cann, the new honorary secretary, will not be content to ask for a subscription from them. The future must be looked to, and the way to secure that, as is being done in the most active centres of hospital energy, Cardiff and North Staffordshire, for instance, is to organise sectional subscriptions. What is wanted is that the men in the munition works should be asked to contribute a small sum per head per week, and that the employers should be asked to contribute, not a casual cheque, but also a sum based on the number of their employees. It is very important to let this step be generally taken. The custom should be formed before the days of reconstruction so that it may be naturally applied after the war to the industries to which the munition workers will return.

### THE EXTENSIONS AT EALING HOSPITAL.

Thanks to the Ealing Chamber of Commerce, a new casualty room and a new $x$-ray room have been provided at Ealing Hospital. The first is indeed a small unit, designed by Mr. Hall Jones, which will be connected by a covered way with the main institution. The old casualty room has been converted into an $x$-ray room, the installation for which has been supplied by Messrs. Newton and Wright, of Wigmore Street. The hospital, which has only been at work for seven years. is steadily growing, and now includes among its patients discharged soldiers and war pensioners.

### COMPLAINTS AGAINST A MEDICAL BOARD.

For a considerable time the Lambeth Tribunal have been dissatisfied with the examination of men for the Army by the No. 1 Travelling Medical Board of Battersea. In accordance with the instructions of the Tribunal, the Clerk forwarded to the Ministry of National Service early in the year a long list of men who, in the first instance, were passed by this Board for the three higher grades of service, but who had afterwards, as the result of re-examination by the Special or other Medical Boards, been either considerably reduced in classification or rejected altogether. The Tribunal has now received a reply from the Ministry of National Service in which it is stated that a special report was called for in the cases of eight men, seven of whom were totally rejected at Camberwell not long after they had been approved at Battersea. In the cases of four of these men, they were unable to trace their documents. With reference to one man the Travelling Medical Board did not possess the information that the Tuberculosis Officer for Lambeth had found tubercle bacilli in his sputum, whilst in the case of another man additional evidence was placed at the disposal of the Camberwell Board. Another had received an injury to his left eye between the dates of the two examinations, whilst the $x$-rays photograph and report assisted the Camberwell Board, which was not at the disposal of the Battersea Board. This is all very well as far as it goes, but the fact remains that many Medical Boards are loath to weigh any other evidence but their own immediate examination. The Lambeth Tribunal has done well to have the Board's action investigated.

### THE REASONS FOR THE CLASSIFICATIONS.

The Ministry of National Service adds that, "after making all allowances for unavoidable difference of opinion and for symptoms which have developed subsequent to examination by the Travelling Medical Board, the latter appears to have been too reluctant to reject doubtful cases. This attitude is explained in a letter from the Chairman of the Travelling Medical Board, in which he states 'it was felt that to give a man a final and irrevocable discharge was a responsibility which should not be undertaken in the case of any man who either was or was likely to become of service in the Army in any capacity in which he might liberate a more able-bodied man for more active employment.' This Board has now ceased to exist as a Travelling Medical Board, and has been transferred to Camberwell Baths."

### A DRASTIC RECONSTRUCTION DEMANDED.

This reply to the Ministry of National Service has not satisfied the members of the Lambeth Tribunal, who still demand a drastic reconstruction of the Medical Board, which has simply been met by the Department transferring the Travelling Board to Camberwell Baths and altering the name! It still sat under the same presidency as when it was known as No. 1 Travelling Medical Board, but only by a reconstruction could public confidence be restored in the medical examinations conducted at Camberwell Baths. The Tribunal draw the special attention of the Ministry to the admission of the Chairman in his letter that his Medical Board had practically shirked the responsibility of rejecting men from military service, which both the Army Council and the Ministry of National Service had vested in these Boards. Not only had this Board not carried out this responsibility, but it had also in the cases of previously rejected or low categoried men elevated them into the higher categories—a line of action which was condemned by the course subsequently followed by other Medical Boards. The truth, therefore, cannot be shirked, for the Board's record is a poor one.

### THE SEAMEN'S HOSPITAL, MARSEILLES.

Lady Burghclere and a committee are forming a scheme to provide a seamen's hospital at Marseilles. A sum of £20,000 is required, to which a good start has been given by the shipping industry. The proposed hospital will be called the Marseilles British Merchant Seamen's Hospital. Much is hoped from King George's Fund if the trustees are satisfied that the rules which allow them to make grants to marine benevolent institutions throughout the kingdom will not be infringed by aiding a hospital which, though a British institution, is to be situated on the shores of the Mediterranean. There seems little reason to fear that all the money required will not be quickly subscribed.

### A NEW CHILDREN'S HOSPITAL.

The urgent need for an up-to-date children's hospital in connection with Kidderminster Infirmary was taken a step nearer satisfaction by an announcement made by Dr. Lionel Stretton at the recent annual meeting. He read a letter from Mr. Stanley Baldwin, M.P. for West Worcestershire, and Junior Lord of the Treasury, in which Mr. Baldwin wrote: "I have instructed the Bank of England to prepare a transfer of £5,000 5 per cent. War Loan out of my name into those of the four trustees named in Mr. Chambers' letter. I wish the income to be used for the general service of the infirmary, and when opportunity arises for building a new hospital, the capital to be applied to that object." This generous gift should attract others, so that the Building Fund may be complete as soon as building can be undertaken.

### THE GIFT OF A MEAL.

THE Keighley Victoria Hospital, thanks to the activities of its Comforts Committee, has received for its military patients the gift of meat or fish breakfasts twice a week. Another gentleman has provided a weekly meat dinner, a lady a meat breakfast once a week; while tea both on Tuesday and also on Friday has been provided by two ladies. What a pity that the restriction of supplies does not enable hospitals to appeal successfully for gifts of meals for all their patients! It would probably be at least as popular and quite as useful as the gifts in kind on which many have come to rely to keep their housekeeping expenses within something like reasonable bounds. To beg for a breakfast could hardly be less successful than to beg for "the funds."

### THE ORGANISATION OF OUTLYING DISTRICTS.

THE Royal Eye and Ear Hospital, Bradford, is another institution which is feeling the pinch created by the carelessness of outlying districts which are content to send cases unaccompanied by subscriptions. A recent analysis made by the finance committee showed that the outlying districts did not contribute more than one-half of the cost which their cases received in treatment. Mr. E. R. Firth lately went to the extreme length of publicly naming the laggard districts. The chairman of the Bradford Hospital Fund, Mr. Herbert Gill, remarked that in the case of one district which showed an improvement in this respect this had been due to the efforts of the committee through the amalgamation of charity committees. Here, again, the moral is too plain to be missed.

### THE FIGHT AGAINST ANTHRAX.

SOME interesting facts were revealed at the annual meeting of the Anthrax Investigation Board, held a few days ago under the chairmanship of Mr. George Ackroyd. During the past year no fewer than 1,090 samples of material had been tested for the bacillus, and cultivations were obtained from 103; of these, fifty-five had been sent for investigation arising out of actual cases of the disease. Out of the number of positives, eighty-six were from samples of wool or hair that did not have the danger-signal of blood upon them, most of these apparently coming from "anthrax districts." In Bradford itself there were thirteen cases, of which six died, and in the surrounding districts thirty-five, twenty-eight making a good recovery. The great importance of an early diagnosis was emphasised; in only two of the six fatal cases was a diagnosis made at an early stage, and in some no diagnosis at all was arrived at. It was urged that in any doubtful cases the services of an anthrax expert should at once be enlisted, more especially as anthrax of the lungs or viscera were both hard to diagnose and rapidly fatal.

### THE STANDARDISATION OF SALVARSAN SUBSTITUTES.

A SPECIAL committee has been appointed by the Medical Research Committee to consider the methods of testing and manufacture of salvarsan and its many substitutes, and the methods and results of their clinical administration. The cutting off of all supplies of the German product in 1914 led to the market being flooded with all manner of drugs of unknown composition, many purporting to be identical with, or superior to, the original article. That there were some untoward results from their injection is well known. By now surgeons have separated out the reliable products and discarded the remainder. The committee will be of the greatest value to the general practitioner if it is able to give a concise and authoritative *résumé* of recent experience and a summary of modern technical methods. The chairman is Surgeon-General H. D. Rolleston. Other members are Lieut.-Col. L. W. Harrison and Professor William Bulloch.

### FOOD AND THE INVALID.

FOR the guidance of hospital committees and private practitioners called upon to give medical certificates for extra allowances of rationed articles, the Ministry of Food, on the advice of a committee of reference of the Colleges of Surgeons and Physicians and the Central Medical War Committee, has drawn up a list of diseases and the special foods to be allowed in each. Thus sugar can only be allowed in cases of dysphagia, cancer of tongue, œsophagus, etc., and wounds of the jaw. Extra meat and fat can be allowed in cases of diabetes and tuberculosis, cœliac disease, and pancreatic insufficiency. Milk can be supplied in any case of acute illness, to suckling and pregnant women, and to consumptives. But certificates can only be granted for periods of one week, unless special permission has been obtained from the local food office. It is interesting to note that the committee consider there is no disease for which cream should be allowed, and that there is no evidence that the present "war bread" is in any way injurious to health, nor that any malady requires bread made from superior flour.

### THE THIRD LONDON "GAZETTE."

IN these days of change it is pleasant to see the regular contributors to the *Gazette* of the 3rd London General Hospital, Wandsworth, still writing in its pages. The Easter number, excellently illustrated by Sergeant Noel Irving, Private Hemsley, Sergeant Vernon Loriner and Lance-Corporal J. H. Dowd and others, has several good stories. These it would be a shame to quote, besides half the popularity of this *Gazette* has been gained through its illustrations. These are still excellent, and it is well to note that the letterpress on the whole is on a level with them.

### TO OUR READERS.

**CONTRIBUTIONS are specially invited from any of our readers to these columns. They should deal with topical subjects and news. They must be authenticated for the information of the Editor only. The minimum payment if published is 5s. There is no hard-and-fast rule as to space, but notes of about twenty lines in length are preferred.**

